# st2traj: deconvolution-informed trajectory inference for multi-timepoint spatial transcriptomics

**DOI:** 10.1093/bioinformatics/btag645

**Published:** 2026-08-27

**Authors:** Zhuo Wang, Chiping Zhang

**Affiliations:** Department of Computational Mathematics and Cybernetics, Shenzhen MSU–BIT University, Shenzhen, Guangdong 518172, P.R. China; School of Mathematics, Harbin Institute of Technology, Harbin, Heilongjiang 150000, P.R. China

## Abstract

**Motivation:**

Multi-timepoint spatial transcriptomics enables study of developmental processes in native tissue context, but cell-state mixtures within spots and lack of direct spatial correspondence across sections complicate trajectory inference and biological interpretation.

**Results:**

st2traj is a deconvolution-informed trajectory framework using spot-state composition for multi-timepoint spatial trajectory inference. In a human heart pseudo-spot benchmark, DECODE showed competitive and balanced performance among five deconvolution methods. In multi-timepoint human heart data, unscaled DECODE-derived proportions produced smoother trajectory fields and stronger agreement with expression-derived marker programs than normalized spot-level expression. st2traj also showed greater spatial coherence than spaTrack, while exploratory comparisons with moscot and CASCAT revealed complementary method-specific strengths. Application to an independent chicken heart dataset recovered stage-associated trajectory changes across D7, D10, and D14.

**Availability and Implementation:**

Source code: https://github.com/xiaoxiaoxier/st2traj. Software v0.1.0 and processed data are archived at Zenodo: https://doi.org/10.5281/zenodo.21487030 and https://doi.org/10.5281/zenodo.21502094.

## 1 Introduction

Spatial transcriptomics has enabled transcriptome-wide profiling in intact tissue while preserving spatial context, creating new opportunities to study how molecular states are organized across development, homeostasis, and disease (Ståhl *et al.* 2016, Rodriques *et al.* 2019). Beyond single-slice analysis, multi-timepoint spatial transcriptomics is especially promising for investigating dynamic biological processes, because it captures coordinated transcriptomic changes together with tissue-level spatial reorganization across developmental or temporal stages (Asp *et al.* 2019, Mantri *et al.* 2021). These datasets are conceptually well suited for trajectory analysis, but they also pose challenges that are distinct from those encountered in conventional single-cell trajectory inference. A major difficulty is that widely used sequencing-based spatial platforms, such as 10x Visium, measure spots that often contain mixtures of multiple cell states rather than single cells (Maynard *et al.* 2021, Janesick *et al.* 2023). As a result, direct trajectory inference on spot-level gene expression can be difficult to interpret biologically. The inferred structure may reflect compositional mixing, local transcriptional variation, and section-specific effects simultaneously (Cable *et al.* 2022, Kleshchevnikov *et al.* 2022). In addition, spatial coordinates are not directly comparable across tissue sections because slices may differ by translation, rotation, or morphology, further complicating trajectory inference across time (Yu and Xie 2025, Huizing *et al.* 2026). Classical pseudotime and velocity methods were primarily developed for single-cell transcriptomics (Trapnell *et al.* 2014, La Manno *et al.* 2018, Bergen *et al.* 2020), and more recent spatiotemporal approaches, including optimal-transport-based methods, have begun to address spatial trajectory inference through time (Schiebinger *et al.* 2019, Klein *et al.* 2025, Shen *et al.* 2025, Huizing *et al.* 2026, Peng *et al.* 2026). However, for spot-based multi-timepoint spatial transcriptomics, the choice of representation used for trajectory inference remains insufficiently explored.

Cell type deconvolution offers a natural way to address spot mixing by estimating spot-level cellular or state composition from a matched single-cell reference. Several deconvolution methods, including DECODE, SPOTlight, RCTD, cell2location, and SpaDAMA, have shown strong performance for recovering spot composition in spatial transcriptomics (Cable *et al.* 2022, Kleshchevnikov *et al.* 2022, Huang *et al.* 2025, Saqib and Kim 2025, Zhao *et al.* 2026). Related spatial transcriptomics tools, including SpaMask, SpaBatch, mclSTExp, and SpaICL, address complementary problems such as spatial representation learning, cross-section correction, expression prediction, and spatial-domain identification (Min *et al.* 2024, 2025, Niu *et al.* 2025, Zhao and Min 2025). At the same time, recent trajectory methods for spatial transcriptomics, such as STORIES, highlight the value of learning interpretable quantities such as a trajectory potential and a potential-derived flow field (Huizing *et al.* 2026). These observations suggest a two-stage strategy for multi-timepoint spot-based spatial transcriptomics: first estimate spot-level state composition, and then infer trajectories on a shared deconvolution-derived state manifold. Despite its intuitive appeal, this representation strategy has not been systematically evaluated for multi-timepoint spot-based trajectory analysis.

Here, we present st2traj, a deconvolution-informed trajectory analysis workflow for multi-timepoint spatial transcriptomics. The central design of st2traj is to use estimated spot-state composition as a common representation across tissue sections and developmental stages, thereby reducing the direct influence of high-dimensional gene-level noise and avoiding the need for spatial coordinate correspondence between sections. In the current implementation, st2traj estimates spot-level cell-state compositions from a user-provided single-cell reference, organizes spots in a shared composition-aware representation, and applies STORIES-based trajectory inference to estimate trajectory potential and directional flow.

We systematically evaluate the main design choices of this workflow. Using a human heart pseudo-spot benchmark, we compare DECODE with SPOTlight, RCTD, cell2location, and SpaDAMA and retain DECODE as a competitive and practical default spot-state estimation backend for the current implementation. We then compare expression-based and deconvolution-derived trajectory representations and select unscaled DECODE-derived proportions as the default representation based on spatial coherence and agreement with marker programs calculated from normalized spot-level expression. We also examine the sensitivity of downstream trajectory inference to perturbations in the estimated spot-state compositions.

The workflow is evaluated in a multi-timepoint human heart case study through tissue-level spatial patterns, expression-derived marker programs, and comparisons with spaTrack. Exploratory analyses with moscot and CASCAT provide additional comparisons with optimal-transport and spatial pseudotime approaches (Klein *et al.* 2025, Shen *et al.* 2025). Finally, application to an independent developmental chicken heart dataset evaluates the framework in a different species and with a different cell-state annotation scheme. Together, these analyses support st2traj as a practical and flexible framework for deconvolution-informed trajectory analysis of multi-timepoint spatial transcriptomics data.

## 2 Materials and methods

### 2.1 Overview and problem formulation

st2traj is a deconvolution-informed trajectory analysis framework for multi-timepoint spatial transcriptomics. In the current implementation, DECODE is used to estimate spot-level cell-state proportions, and STORIES is applied to the resulting spot-by-state representation to infer trajectory potential and directional flow. Developmental-stage labels were retained for trajectory orientation and stage-level summaries. Spatial coordinates were standardized separately within each tissue section and used by the trajectory backend for spatial regularization, while the original coordinates were retained for tissue-level projection and spatial evaluation. Direct coordinate correspondence between tissue sections was not required.

We consider a multi-timepoint spatial transcriptomics dataset containing *n* spots collected across *T* developmental time points and one or more tissue sections. For each spot i∈{1,…,n}, let $x_{i}\in R^{G}$ denote its gene-expression vector across *G* genes, $s_{i}\in R^{2}$ its spatial coordinate within the corresponding section, and $t_{i}\in \{1,\ldots ,T\}$ its developmental time-point label. Because each spatial spot may contain multiple cell states, st2traj first represents each spot by its estimated state composition. The number of coarse states, *K*, is determined by the annotation of the user-provided single-cell reference and is not fixed by the algorithm. For the human heart dataset, the seven coarse states were cardiomyocytes (CM), endothelial cells (EC), epicardial cells (EpC), fibroblasts and epicardium-derived cells (FB/EPDC), immune cells, neural cells, and smooth muscle cells, mural cells, and pericytes (SMC/MC/PC). For the chicken heart dataset, the six dataset-specific states were cardiomyocytes (CM), endothelial and endocardial cells (EC/ENDO), epicardial cells (EPI), erythroid cells, smooth muscle cells, mural cells, and pericytes (SMC/MC/PC), and valve cells, fibroblasts, and epicardium-derived cells (VALVE/FB/EPDC).

For each spot *i*, DECODE returns a composition vector


$$p_{i}={\left(p_{i1},\ldots ,p_{iK}\right)}^{⊤},$$

where $p_{ik}$ is the estimated proportion of state *k* in spot *i*. The proportions satisfy


$$p_{ik}\geq 0,\;\sum_{k=1}^{K}p_{ik}=1.$$

The spot-level vectors are combined into the composition matrix


$$\begin{array}{c} P=\left[\begin{array}{c} p_{1}^{⊤}\\ \vdots \\ p_{n}^{⊤} \end{array}\right]\in R^{n\times K}, \end{array}$$

which provides a shared representation across all tissue sections.

Based on this representation, st2traj infers for each spot a scalar trajectory potential


$$\phi_{i}\in R,$$

together with a directional field on the learned state manifold. The potential $\phi_{i}$ describes the relative position of a spot along the inferred developmental trajectory.

### 2.2 Data preprocessing and pseudo-spot benchmark

The human heart single-cell RNA-sequencing reference was restricted to developmental ages 8, 10, and 12 weeks. Fine-grained cell annotations were mapped to seven coarse states: CM, EC, EpC, FB/EPDC, Immune, Neural, and SMC/MC/PC. Cells without a valid coarse-state label were removed, and at most 500 cells were randomly retained from each state to reduce class imbalance. Biological samples, rather than individual cells, were randomly divided into model-development and held-out test groups at an 80:20 ratio. Training and validation pseudo-spots were generated exclusively from the model-development samples, whereas test pseudo-spots were generated exclusively from the held-out samples. Only cell states represented in both partitions were retained. Raw RNA counts were used to construct the pseudo-spots. All evaluated methods were provided with identical pseudo-spot count matrices, ground-truth composition matrices, and input genes. Method-specific normalization, feature selection, and model fitting were subsequently performed according to the requirements of each method.

For each pseudo-spot *m*, the total number of contributing cells was sampled as


$$N_{m}∼\mathrm{Uniform}\{10,\ldots ,30\}.$$

A target composition was sampled from a symmetric Dirichlet distribution,


$$\pi_{m}∼\mathrm{Dirichlet}({1\;}_{K}),$$

and the numbers of cells drawn from the K=7 states were sampled as


$$(n_{m1},\ldots ,n_{mK})∼\mathrm{Multinomial}(N_{m},\pi_{m}).$$

Cells were then sampled from the corresponding state-specific pools, and their raw count vectors were summed to obtain the pseudo-spot count profile. No additional sequencing-depth or dropout perturbation was introduced. The ground-truth proportion of state *k* in pseudo-spot *m* was defined as


$$q_{mk}=\frac{n_{mk}}{N_{m}}.$$

We generated 1200 training pseudo-spots, 300 validation pseudo-spots, and 300 held-out test pseudo-spots. The validation set was used for model and parameter selection, whereas the test set was used only for final evaluation. Pseudo-spot generation used random seed 42, and the final DECODE model was trained using random seed 2021.

The benchmark included DECODE, SpaDAMA, cell2location, SPOTlight, and RCTD. RCTD was run in full mode because the simulated pseudo-spots could contain more than two cell states. Doublet mode was evaluated separately as a sensitivity analysis. For each method, let


$${\hat{q}}_{m}={\left({\hat{q}}_{m1},\ldots ,{\hat{q}}_{mK}\right)}^{⊤}$$

denote the predicted composition of pseudo-spot *m*. For state *k*, the root mean squared error was


$${\text{RMSE}}_{k}=\sqrt{\frac{1}{M}\sum_{m=1}^{M}{\left({\hat{q}}_{mk}-q_{mk}\right)}^{2}},$$

where *M* is the number of test pseudo-spots. Pearson correlation was calculated as


$${\text{Corr}}_{k}=\mathrm{corr}({\hat{q}}_{\cdot k},q_{\cdot k}),$$

and the concordance correlation coefficient was


$${\text{CCC}}_{k}=\frac{2\;\mathrm{cov}({\hat{q}}_{\cdot k},q_{\cdot k})}{\sigma_{{\hat{q}}_{\cdot k}}^{2}+\sigma_{q_{\cdot k}}^{2}+{\left(\mu_{{\hat{q}}_{\cdot k}}-\mu_{q_{\cdot k}}\right)}^{2}}.$$

The three metrics were calculated separately for each of the seven states. Overall CCC, Pearson correlation, and RMSE were calculated after flattening the M×K ground-truth and predicted composition matrices into paired vectors. Confidence intervals for the overall metrics were estimated using 2000 spot-level bootstrap replicates.

For the real human heart Visium analysis, raw spot count matrices were loaded together with sample identifiers, developmental-stage labels, and spatial coordinates. For DECODE inference, genes in each section were reordered to match the gene list used during model training. Genes missing from a spatial section were assigned zero counts, whereas genes absent from the training reference were excluded. No explicit batch correction was performed before DECODE inference. For the expression-based trajectory comparison, the analysis was restricted to genes shared across the three Visium sections. Counts were library-size normalized to 10 000 counts per spot and log1p-transformed. Up to 3000 highly variable genes were selected while accounting for developmental stage, after which the expression matrix was scaled with values clipped at 10. The first 20 principal components were used as the expression-based input for trajectory inference. The original sample identities, developmental stages, and spatial coordinates were retained for downstream analysis and visualization.

### 2.3 Spot-state estimation and representation construction

Following the pseudo-spot benchmark, DECODE was retained as the default spot-state estimation backend in the current implementation of st2traj. For each spatial transcriptomics section, the spot-level count matrix was matched to the gene order used during DECODE training and supplied to the trained model. The resulting *K*-dimensional composition vector $p_{i}$ was stored for each spot together with its sample identity, developmental-stage label, and spatial coordinates in an AnnData object. Three candidate representations were considered for downstream trajectory inference.

The expression-based representation consisted of the first 20 principal-component scores obtained from the preprocessed spot-expression matrix described above:


$$r_{i}^{\left(\text{expr}\right)}=u_{i},\;u_{i}\in R^{20}.$$

The unscaled DECODE representation used the inferred composition vector directly:


$$r_{i}^{\left(\text{decode}\right)}=p_{i}.$$

For comparison, a feature-wise scaled DECODE representation was also constructed:


$$r_{i}^{\left(\text{decode}\;\text{scaled}\right)}=D^{-1}(p_{i}-\mu ),$$

where $\mu \in R^{K}$ contains the mean proportion of each state across all analyzed spots, and *D* is the diagonal matrix of the corresponding standard deviations. For a selected representation, the trajectory input matrix was


$$\begin{array}{c} R=\left[\begin{array}{c} r_{1}^{⊤}\\ \vdots \\ r_{n}^{⊤} \end{array}\right]\in R^{n\times d_{R}}, \end{array}$$

where $d_{R}=20$ for the expression-based representation and $d_{R}=K$ for the two DECODE-derived representations.

All three representations were constructed using the same spots, sample identities, and developmental-stage labels, so that the comparison reflected only differences in the trajectory input representation. Based on this comparison, unscaled DECODE-derived proportions were used as the default representation in the subsequent human and chicken heart analyses. The number of states *K* was determined by the dataset-specific single-cell annotation and was not fixed by the software.

### 2.4 Trajectory inference and spatial summaries

STORIES (Huizing *et al.* 2026) was used as the trajectory-inference backend. The selected representation was supplied directly to STORIES: the expression-based analysis used the 20-dimensional principal-component representation described above, whereas the DECODE-based analyses used the corresponding *K*-dimensional composition representation. Spatial coordinates were centered and scaled separately within each tissue section and used by STORIES for spatial regularization. The original coordinates were retained for tissue-level visualization and spatial evaluation. STORIES was fitted using a learning rate of $5\times 10^{-3}$ and a quadratic regularization weight of $5\times 10^{-4}$. For each spot *i*, STORIES returned a scalar trajectory potential $\phi_{i}$ and a directional field $v_{i}$. The potential describes the relative position of a spot along the inferred trajectory and should not be interpreted as absolute developmental time. Because the sign of the potential is arbitrary, it was oriented so that its Spearman correlation with the ordered developmental stages was non-negative. This operation changes only the sign and does not alter spot ranking or spatial smoothness. For visualization, a separate two-dimensional UMAP was constructed for each representation using n_neighbors = 15, min_dist = 0.5, Euclidean distance, and random seed 2021. UMAP was used only for visualization and did not affect trajectory inference.

Potential values were standardized separately within each tissue section for comparisons of local spatial patterns. For visualizations requiring a common scale across developmental stages, the potential was instead standardized across all spots. Standardized values were mapped back to the original tissue coordinates. The dominant state of spot *i* was defined as


$$d_{i}=\text{arg}\underset{k\in \{1,\ldots ,K\}}{\max}p_{ik}.$$

Internal consistency between trajectory potential and DECODE-derived composition was evaluated using Spearman correlations calculated separately within each developmental stage. For state *k*, the stage-balanced correlation and overall summary were defined as


$${\bar{\rho }}_{k}=\frac{1}{T}\sum_{t=1}^{T}{\mathrm{corr}}_{S}\left(\phi^{\left(t\right)},p_{\cdot k}^{\left(t\right)}\right),\;C_{\text{comp}}=\frac{1}{K}\sum_{k=1}^{K}|{\bar{\rho }}_{k}|.$$

Because the trajectory input and evaluated proportions were both derived from DECODE, these statistics were interpreted as internal representation-consistency measures rather than independent biological validation.

Spatial coherence was evaluated separately within each tissue section using a symmetrized six-nearest-neighbor graph constructed from the original spatial coordinates. Spatial roughness was defined as the mean absolute difference in section-standardized potential between neighboring spots:


$$S_{t}=\frac{1}{\left|E_{t}\right|}\sum_{(i,j)\in E_{t}}\left|z_{i}^{\left(\text{section}\right)}-z_{j}^{\left(\text{section}\right)}\right|,$$

where $E_{t}$ denotes the unique spatial-neighbor pairs in section *t*. Lower values indicate smoother spatial patterns. Moran’s *I* was additionally calculated using row-standardized weights on the same spatial graph, with larger positive values indicating stronger spatial autocorrelation.

### 2.5 Human heart analysis, external comparisons, and assessment

We applied st2traj jointly to three human heart spatial transcriptomics sections from developmental stages w8, w10, and w12: V10A13-157_A1, V10F24-105_A1, and V10T03-307_D1, respectively. DECODE-derived spot-state proportions were used for trajectory inference, while developmental-stage labels and original spatial coordinates were retained for trajectory orientation, spatial projection, and evaluation. We compared st2traj with spaTrack on the same spots. Because spaTrack estimates section-wise pseudotime from expression, whereas st2traj estimates a joint potential from spot-state composition, the methods were compared using spatial coherence and expression-derived marker-program associations rather than absolute trajectory values. The spaTrack pseudotime was oriented using the same developmental-stage criterion as the st2traj potential and standardized separately within each section. Spatial coherence was evaluated using Moran’s *I*, and marker agreement was evaluated using within-stage Spearman correlations.

For an exploratory optimal-transport comparison, moscot (Klein *et al.* 2025) was applied to the unscaled DECODE-derived composition representation. A SpatioTemporalProblem was constructed using composition vectors as the joint feature space and original tissue coordinates as the spatial feature space. Transport couplings between w8–w10 and w10–w12 were estimated using α=0.5, ε=0.01, and rank 100. Because moscot returns transport couplings rather than pseudotime, an OT-informed progression score was derived by projecting each spot and its barycentric transported future position onto the developmental axis from w8 to w12 on the common DECODE-derived UMAP. The current and future projections were averaged for w8 and w10 spots, whereas only the current projection was used for w12 spots. The resulting score was oriented toward increasing developmental stage and standardized within each stage. It was used only for exploratory comparison and was not treated as a native moscot pseudotime. CASCAT (Yu *et al.* 2025) was applied to the representative w10 section using a prespecified CM-associated root spot. CASCAT pseudotime and the st2traj potential were standardized within w10 and compared using the same six-nearest-neighbor spatial graph and expression-derived marker-module scores. The moscot and CASCAT analyses were treated as exploratory because their inputs and outputs differ from those of st2traj.

Sensitivity to uncertainty in the DECODE-derived compositions was evaluated by sampling


qi*∼Dirichlet(1 K)

and defining


$$p_{i}^{\left(\epsilon \right)}=(1-\epsilon )p_{i}+\epsilon q_{i}^{*}.$$

Perturbation levels ϵ∈{0,0.05,0.10,0.20,0.30} were evaluated. For each non-zero level, 10 independently perturbed composition matrices were generated and the complete trajectory model was refitted. Trajectory stability was measured by the Spearman correlation with the baseline potential, spatial roughness by the mean neighboring-score difference, and marker-program retention by correlations with expression-derived marker modules. Results were summarized using the median and the 10th–90th percentiles across replicates.

Seven marker modules were defined for CM, EC, EpC, FB/EPDC, Immune, Neural, and SMC/MC/PC using the genes listed in Table S1. Raw counts were normalized to 10 000 counts per spot and log1p-transformed separately within each tissue section. Module scores were calculated using scanpy.tl.score_genes with use_raw = False and random seed 0. Modules with fewer than three available genes were not evaluated. Marker-module scores were not used as trajectory inputs. Spearman correlations between trajectory scores and marker modules were calculated separately within w8, w10, and w12 and then averaged equally across stages. All available modules were included in quantitative comparisons, while CM, EC, FB/EPDC, and SMC/MC/PC were selected for detailed visualization. Marker trends were additionally examined across within-stage potential deciles and between the bottom and top 20% of potential values.

### 2.6 Cross-species evaluation in chicken heart

We applied st2traj to the developmental chicken heart dataset GSE149457 (Mantri *et al.* 2021), including matched single-cell RNA-sequencing and spatial transcriptomics data from D7, D10, and D14. The D4 section was not included. Single-cell annotations were mapped to six dataset-specific coarse states: CM, EC/ENDO, EPI, Erythroid, SMC/MC/PC, and VALVE/FB/EPDC. A shared feature space of 3007 highly variable genes present in the single-cell reference and all three spatial sections was used. Raw counts were retained for DECODE training and prediction. We generated 6000 training pseudo-spots, 300 validation pseudo-spots, and 1000 held-out test pseudo-spots, with 20 single cells mixed in each pseudo-spot. DECODE was trained using a batch size of 4 and a maximum of 200 epochs.

The trained model was applied separately to D7, D10, and D14, and the resulting six-state composition vectors were combined for trajectory analysis. Trajectory inference, potential orientation, spatial projection, and state–potential associations followed the human heart analysis. Association between potential and developmental day was evaluated using Spearman correlation, and differences among stages were tested using the Kruskal–Wallis test.

### 2.7 Software implementation and reproducibility

st2traj was implemented in Python and uses AnnData objects to store expression data, spot-state compositions, sample and time-point labels, spatial coordinates, and trajectory outputs. DECODE and STORIES were used as the current spot-state estimation and trajectory-inference backends, respectively. State columns are detected automatically, so the workflow does not require a fixed number or identity of states. The human heart workflow, comprising 6507 spatial spots, was benchmarked on a Linux workstation with 32 logical CPU cores and one NVIDIA GeForce RTX 4080 SUPER GPU with 16 GB of memory. DECODE training, prediction, representation construction, and STORIES-based trajectory inference required 78.51 s, 1.93 s, 9.49 s, and 1221.02 s, respectively, resulting in a total runtime of 1310.95 s (21.85 min) for the four main computational steps. A single CUDA-capable GPU was used, and multi-GPU computing was not required.

## 3 Results

### 3.1 Overview of the st2traj workflow

**Figure 1:**
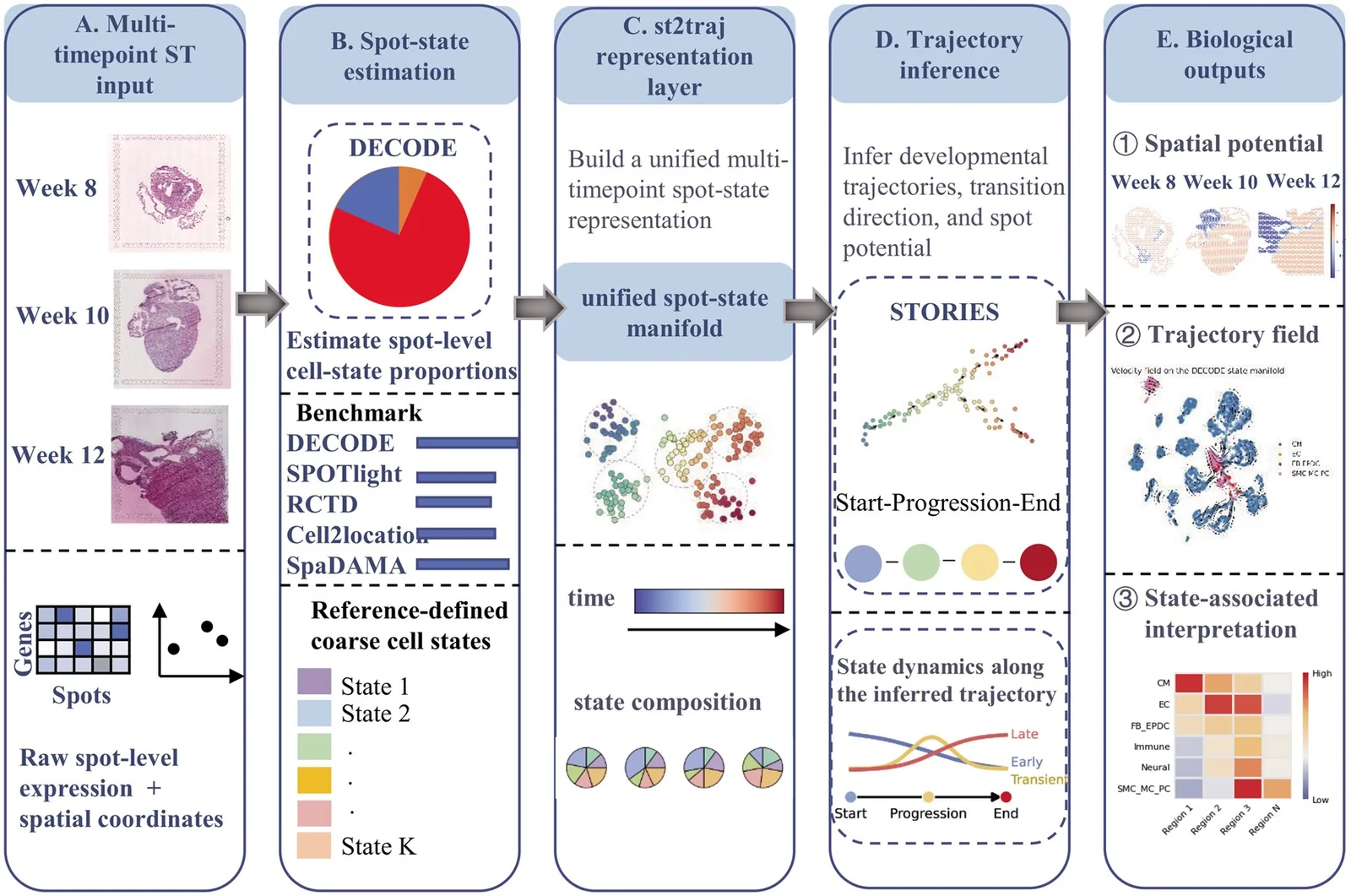
Overview of the st2traj workflow. st2traj is a deconvolution-informed trajectory analysis workflow for multi-timepoint spatial transcriptomics. (A) Multi-timepoint spatial sections are provided with spot-level expression, spatial coordinates, and time-point metadata. (B) Reference-guided spot-state estimation produces coarse state proportions for each spot. (C) The estimated proportions are assembled into a common composition-aware representation across sections. (D) Trajectory inference on this representation yields spot-level potential and a manifold-level directional field. (E) The inferred trajectory is examined on the integrated manifold and in the original tissue coordinates.

st2traj addresses the mixed-cell nature of spot-based spatial transcriptomics by separating spot-state estimation from downstream trajectory inference (Fig. 1). Direct analysis of spot-level expression may combine compositional variation, within-state transcriptional changes, and section-specific effects. The workflow therefore first converts each mixed spot into an estimated coarse state-composition vector and then uses these vectors as a common trajectory representation across tissue sections and developmental stages.

In the current implementation, DECODE is used to estimate spot-state proportions and STORIES is used for trajectory inference. DECODE was retained as a competitive and practical default backend following the pseudo-spot benchmark described below. The estimated proportions are assembled across sections while retaining sample identities, developmental-stage labels, and original spatial coordinates. Trajectory inference is performed in the shared composition-aware representation, whereas tissue coordinates are used for spatial projection and interpretation and are not assumed to correspond directly across sections.

The resulting outputs include a scalar trajectory potential for each spot and a directional field on the learned manifold. These outputs can be visualized jointly across developmental stages or mapped back to the original tissue sections. The following analyses evaluate the choice of spot-state estimation backend and trajectory input representation, examine biological and spatial consistency in developing human heart, assess robustness and external-method agreement, and test the workflow in an independent chicken heart dataset.

### 3.2 Pseudo-spot benchmarking supports DECODE as a competitive default spot-state estimation backend

**Figure 2:**
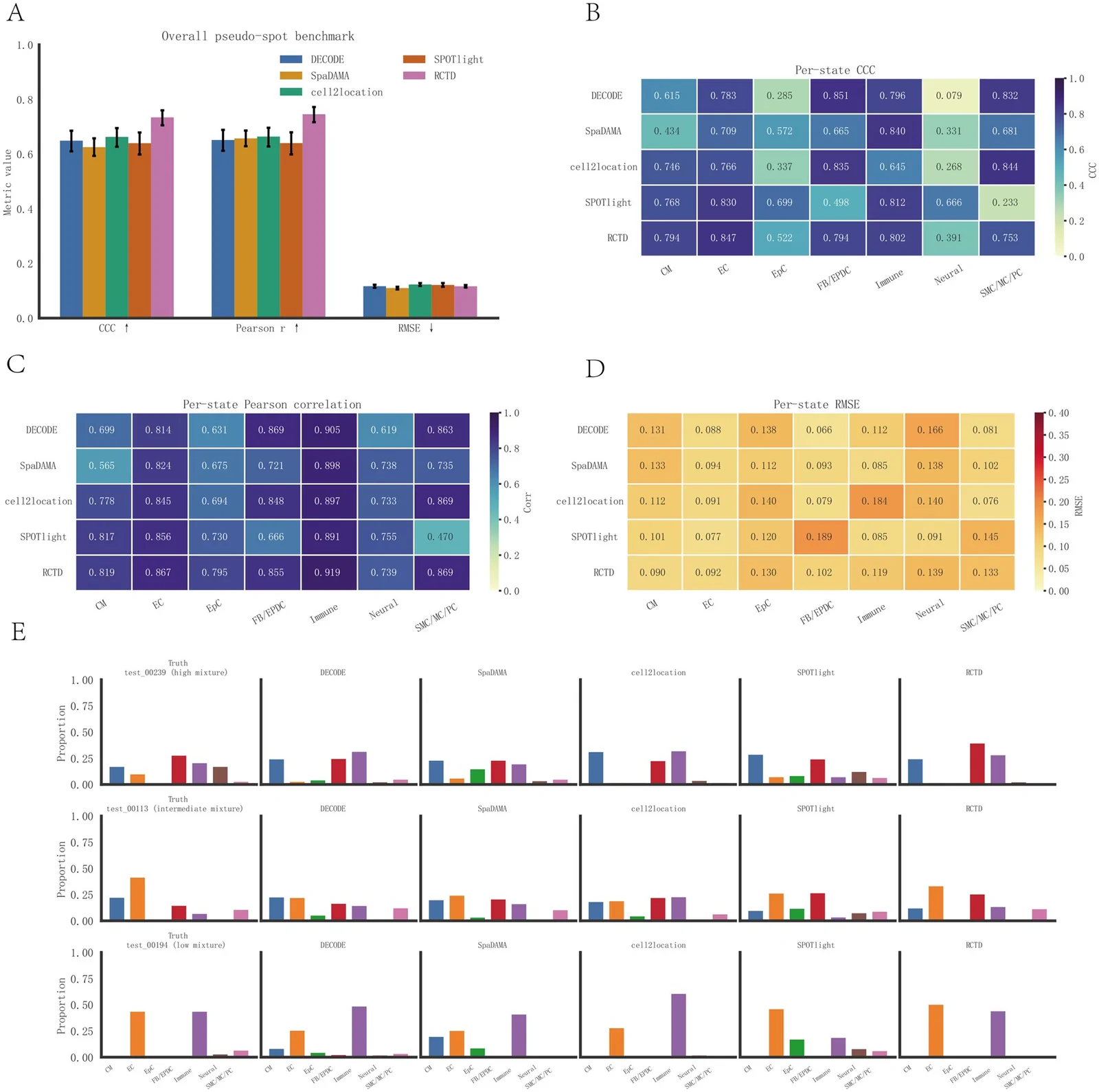
Pseudo-spot benchmark of candidate spot-state estimation methods. DECODE, SpaDAMA, cell2location, SPOTlight, and RCTD were evaluated using the same 300 held-out pseudo-spots and seven coarse cell-state categories. RCTD was run in full mode because the simulated pseudo-spots could contain more than two states. (A) Overall concordance correlation coefficient (CCC), Pearson correlation, and root mean squared error (RMSE). Error bars indicate 95% confidence intervals from 2000 spot-level bootstrap replicates. (B–D) Per-state CCC (B), Pearson correlation (C), and RMSE (D). (E) Representative pseudo-spots with high, intermediate, and low ground-truth composition entropy. Within each entropy stratum, the example closest to the 30th percentile of DECODE spot-wise RMSE was selected.

We evaluated five candidate spot-state estimation methods using pseudo-spots generated from a human heart single-cell reference. The reference was divided by biological sample into model-development and held-out test subsets, and all methods were evaluated on the same 300 test pseudo-spots using identical state definitions, gene space, and evaluation metrics. RCTD-full achieved the highest overall CCC (0.734) and Pearson correlation (0.746), whereas SpaDAMA achieved the lowest RMSE (0.110) (Fig. 2A). The overall CCC, Pearson correlation, and RMSE were 0.649, 0.651, and 0.117 for DECODE; 0.625, 0.657, and 0.110 for SpaDAMA; 0.663, 0.664, and 0.123 for cell2location; 0.640, 0.640, and 0.121 for SPOTlight; and 0.734, 0.746, and 0.116 for RCTD-full, respectively. Per-state performance varied substantially, and no method was uniformly optimal across all seven states (Fig. 2B–D). Representative pseudo-spots further illustrate differences in the predicted composition profiles across methods (Fig. 2E); these examples were selected using predefined composition-entropy and DECODE-error criteria rather than visual inspection alone.

RCTD was additionally evaluated in doublet mode and performed substantially worse than RCTD-full, consistent with the multi-state composition of the simulated pseudo-spots (Fig. S1). This result indicates that RCTD performance depends on whether the assumed mixture mode matches the underlying spot complexity. Although DECODE was not optimal for every metric, its competitive and balanced performance, lack of a required doublet-versus-full mixture specification, and compatibility with the downstream workflow supported its use as the default spot-state estimation backend in the current implementation of st2traj.

### 3.3 Deconvolution-derived representations improve the interpretability of downstream trajectory inference

**Figure 3:**
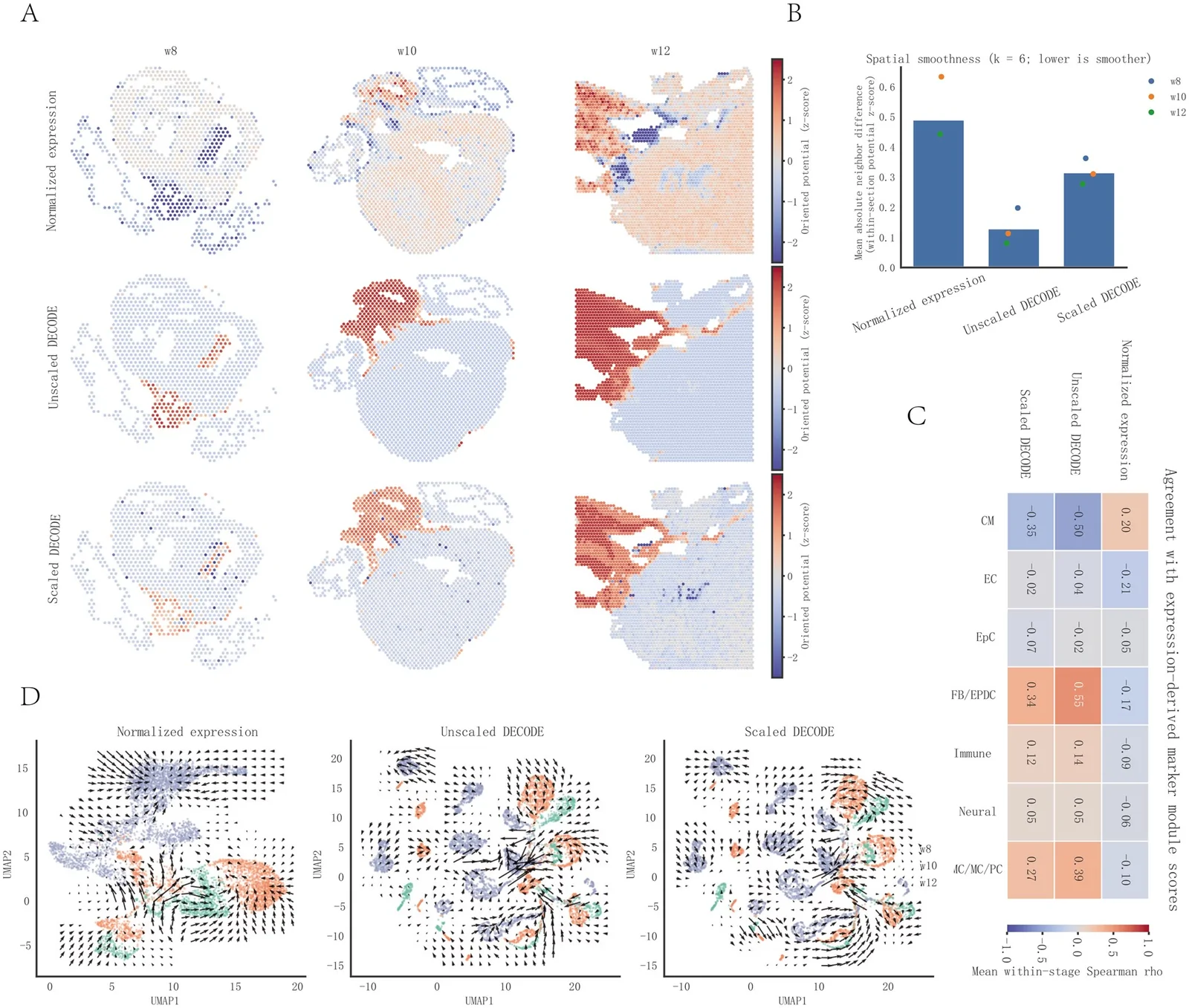
Comparison of expression-based and deconvolution-derived representations for downstream trajectory inference. Three trajectory input representations were compared: normalized spot-level gene expression, unscaled DECODE-derived proportions, and feature-wise scaled DECODE-derived proportions. (A) Spatial maps of consistently oriented trajectory potential across w8, w10, and w12, shown as within-section z-scores. (B) Spatial smoothness, quantified as the mean absolute difference in within-section z-scored potential between each spot and its six nearest spatial neighbors; lower values indicate smoother fields. (C) Stage-balanced signed Spearman correlations between trajectory potential and marker-module scores calculated from normalized spot-level expression. (D) Qualitative comparison of directional fields on the corresponding representation-specific UMAP manifolds. Because the UMAPs were constructed separately, flow organization was interpreted within each representation rather than by direct comparison of manifold geometry.

We next asked whether deconvolution-derived spot representations provided a more suitable input for trajectory inference than normalized spot-level gene expression (Fig. 3). We compared normalized expression, unscaled DECODE-derived proportions, and feature-wise scaled DECODE-derived proportions. The three inferred potentials were oriented using the same predefined criterion before downstream comparison. When projected back to tissue space, the expression-based representation produced greater local heterogeneity, particularly in the w10 and w12 sections, whereas both DECODE-derived representations produced smoother spatial gradients (Fig. 3A). Quantification using neighboring-spot differences confirmed this pattern (Fig. 3B). Unscaled DECODE-derived proportions showed the lowest local score variation, while the scaled representation showed an intermediate level of spatial smoothness.

We next evaluated associations with marker-module scores calculated from normalized spot-level expression (Fig. 3C). These marker scores were not used to construct the DECODE-derived trajectory representations and therefore provided a complementary expression-based assessment. The trajectory inferred from unscaled DECODE proportions showed stronger associations with the CM, FB/EPDC, and SMC/MC/PC programs than the expression-based and scaled DECODE representations, whereas the EC program showed only a weak association. The complementary correlations with DECODE-derived state proportions were treated as internal representation-consistency measurements and are reported separately in Fig. S2B and C. The representation-specific manifolds also showed different qualitative organizations (Fig. 3D). The expression-based manifold appeared more strongly separated by developmental stage, whereas the DECODE-derived representations showed directional organization within the composition-informed state manifolds. Because the UMAP embeddings were constructed independently, their geometries were not compared directly.

Overall, unscaled DECODE-derived proportions provided the best balance of spatial smoothness, expression-derived marker-program agreement, and interpretable manifold-level organization in the present dataset. We therefore used this representation as the default trajectory input for the subsequent analyses.

### 3.4 st2traj reconstructs coherent spatiotemporal state transitions in developing human heart tissue

**Figure 4:**
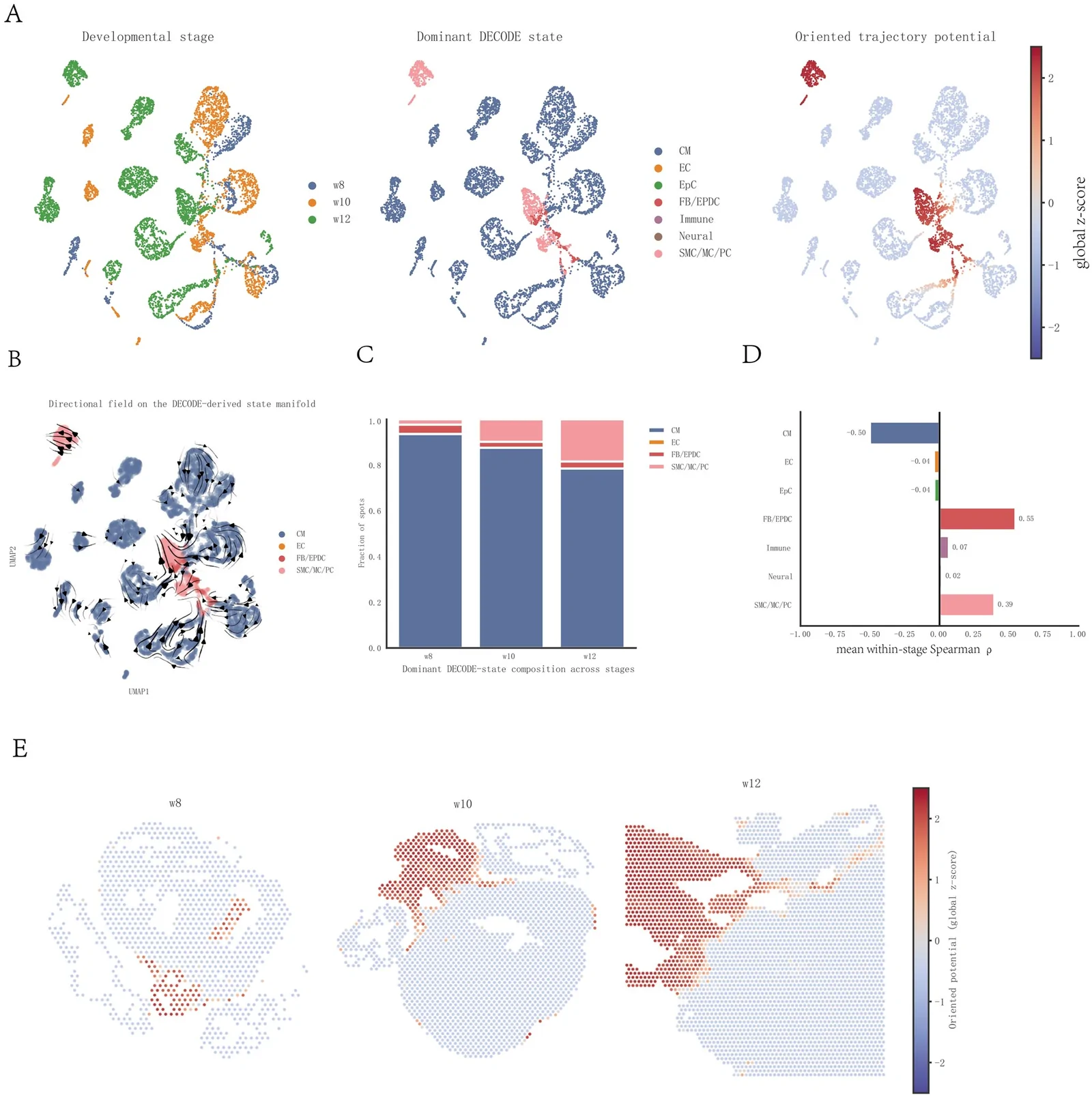
Human heart case study using the final st2traj representation. st2traj was applied to unscaled DECODE-derived proportions from human heart spatial transcriptomics data at w8, w10, and w12. (A) Integrated manifold colored by developmental stage, dominant DECODE state, and globally standardized oriented trajectory potential. (B) Potential-derived directional field on the DECODE-derived state manifold. (C) Fractions of spots assigned to each observed dominant state across developmental stages. (D) Mean within-stage Spearman correlations between trajectory potential and marker-module scores calculated from normalized spot-level expression. (E) Spatial distributions of globally standardized trajectory potential across w8, w10, and w12, shown using a common color scale. Each tissue section is displayed in its original dataset-specific spatial coordinate system. The sections were not anatomically registered, rotated, or reflected across developmental stages.

Using unscaled DECODE-derived proportions as the trajectory input, we applied st2traj to human heart spatial transcriptomics sections from w8, w10, and w12. On the integrated manifold, spots showed structured organization by developmental stage and dominant state, while the oriented trajectory potential varied continuously across partially overlapping stage distributions (Fig. 4A). The potential-derived field showed organized directional structure across the state manifold (Fig. 4B). The fraction of CM-dominant spots decreased across development, whereas FB/EPDC- and SMC/MC/PC-dominant spots became more frequent at later stages (Fig. 4C), indicating remodeling of spot-state composition.

We next evaluated marker-module scores calculated from normalized spot-level expression and not used as trajectory inputs. The CM program was negatively associated with oriented potential (ρ=−0.50), whereas the FB/EPDC and SMC/MC/PC programs showed positive associations (ρ=0.55 and 0.39, respectively); the EC association was weak (ρ=−0.04) (Fig. 4D). Trends across potential, comparisons between low- and high-potential spots, and representative spatial marker maps are shown in Fig. S3. The globally standardized potential formed continuous spatial gradients within the w8, w10, and w12 sections (Fig. 4E). Dominant-state maps and correlations between potential and DECODE-derived proportions are provided in Fig. S2. Because these correlations reuse the DECODE-derived representation, they were interpreted as internal consistency rather than independent biological validation. Together, these results show that the inferred trajectory was associated with coordinated changes in spot-state composition, expression-derived cardiac marker programs, and tissue-level spatial organization.

### 3.5 Spatial coherence, marker-program agreement, and robustness in external comparisons

**Figure 5:**
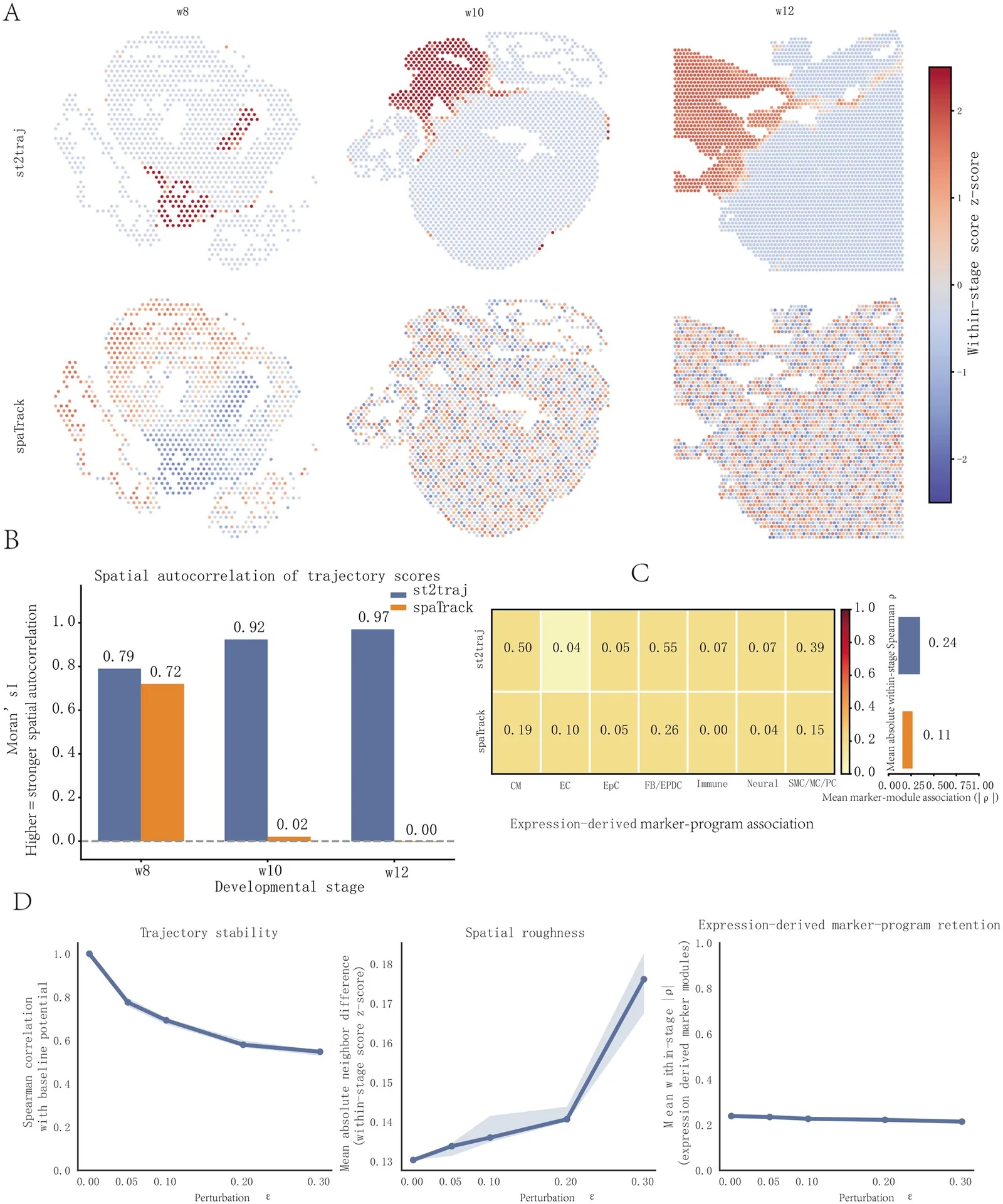
Spatial coherence, expression-derived marker agreement, and robustness of the inferred trajectory. (A) Spatial distributions of within-section standardized st2traj potential and spaTrack pseudotime across w8, w10, and w12. (B) Spatial autocorrelation measured using Moran’s *I*; higher values indicate stronger spatial coherence. (C) Associations between each trajectory score and marker-module scores calculated from normalized spot-level expression. The heatmap shows absolute mean within-stage Spearman correlations, and the summary bars show the mean absolute correlation across marker programs. (D) Sensitivity of st2traj to perturbation of the DECODE-derived composition vectors, evaluated using correlation with the baseline potential, neighboring-score variation, and retention of expression-derived marker associations. For each non-zero perturbation level, the line shows the median and the shaded band shows the 10th–90th percentiles across 10 replicates; ϵ=0 denotes the unperturbed baseline.

To further evaluate st2traj, we compared it with spaTrack on the same multi-timepoint human heart spatial transcriptomics dataset (Fig. 5). Because spaTrack estimates section-wise pseudotime on an expression-based manifold, whereas st2traj infers a common trajectory potential on a deconvolution-derived state manifold, we focused on spatial coherence and agreement with marker programs calculated from normalized spot-level expression. Visual comparison showed that st2traj produced continuous spatial score gradients, whereas spaTrack yielded more locally fragmented patterns, particularly in the w10 and w12 sections (Fig. 5A). Scores from each method were standardized separately within each developmental stage for visualization. Spatial coherence was quantified using Moran’s *I* (Fig. 5B). st2traj showed higher spatial autocorrelation than spaTrack at all three stages, with values of 0.79 versus 0.72 at w8, 0.92 versus 0.02 at w10, and 0.97 versus 0.00 at w12. These results confirmed the greater spatial continuity observed in the tissue maps.

We next compared associations with marker-module scores calculated from normalized spot-level expression (Fig. 5C). st2traj showed stronger associations with the CM, FB/EPDC, and SMC/MC/PC programs than spaTrack. The mean absolute within-stage Spearman correlation across the seven programs was 0.24 for st2traj and 0.11 for spaTrack. Because these marker scores were not used as trajectory inputs, they provided a complementary expression-based assessment. We also evaluated sensitivity to uncertainty in the DECODE-derived compositions by perturbing the estimated proportions and refitting the trajectory model (Fig. 5D). Increasing perturbation reduced correlation with the baseline potential and increased neighboring-score variation, whereas expression-derived marker-program associations declined only gradually. Thus, larger perturbations progressively weakened trajectory stability and spatial coherence, while marker-program associations remained comparatively stable.

The moscot and CASCAT analyses were performed for exploratory comparisons. The moscot analysis was applied to the same DECODE-derived representation across w8, w10, and w12, and an OT-informed progression score was derived from the adjacent-stage transport couplings. Under this score construction, moscot produced lower local spatial roughness and stronger expression-derived marker-program associations than st2traj (Fig. S4). In contrast, st2traj directly provided a potential-derived directional field on the shared spot-state manifold. Because the compared quantities have different definitions, this analysis was treated as an exploratory comparison rather than a direct ranking of trajectory accuracy. CASCAT was additionally applied to the representative w10 section using a prespecified CM-associated root spot. st2traj showed lower neighboring-score variation, whereas the two methods had broadly comparable overall associations with expression-derived marker programs (Fig. S5). This comparison was also considered exploratory because CASCAT and st2traj differ in their input, root-selection, and trajectory-output assumptions. Together, these analyses showed that st2traj produced more spatially coherent scores and stronger expression-derived marker-program agreement than spaTrack in the human heart dataset. The perturbation analysis demonstrated a gradual loss of trajectory stability with increasing composition uncertainty, while the moscot and CASCAT comparisons revealed complementary method-specific strengths.

Furthermore, to assess cross-species applicability, we applied st2traj to an independent chicken heart spatial transcriptomics dataset spanning D7, D10, and D14. The six-state representation produced an organized spot-state manifold, directional field, and spatial potential patterns (Fig. S6A–D). Potential distributions differed significantly among developmental days (Kruskal–Wallis $P=4.77\times 10^{-50}$), although their monotonic association with developmental day was modest (Spearman ρ=0.197; Fig. S6E). State-specific associations with DECODE-derived proportions provided an internal consistency assessment (Fig. S6F). These results support the applicability of the workflow across species and annotation systems.

## 4 Conclusion

We presented st2traj, a deconvolution-informed trajectory analysis workflow for multi-timepoint spatial transcriptomics. In the current implementation, st2traj implements a deconvolution-informed representation strategy, and provides an end-to-end strategy for constructing, evaluating, and interpreting trajectory representations in spot-based spatial transcriptomics data. Through a pseudo-spot benchmark, we retained DECODE as a competitive and practical default spot-state estimation backend, rather than as a universally optimal method. We further showed that deconvolution-derived representations, especially unscaled DECODE-derived proportions, produced smoother spatial trajectory fields and stronger agreement with expression-derived marker programs than normalized spot-level expression. In a multi-timepoint human heart case study, st2traj reconstructed coherent spatial gradients and trajectory patterns associated with changes in dominant spot states and marker programs calculated from normalized spot-level expression. Comparison with spaTrack further showed that st2traj produced greater spatial coherence and stronger expression-derived marker-program agreement in this setting. Exploratory comparisons with moscot and CASCAT revealed complementary method-specific strengths, while application to an independent developmental chicken heart dataset supported the applicability of the workflow across species and annotation systems. Together, these results support the use of deconvolution-informed state representations as a practical basis for trajectory inference in spot-based multi-timepoint spatial transcriptomics.

This study also has several limitations. First, although the software separates representation construction from the backend methods, the present evaluation focused primarily on the DECODE–STORIES configuration. Second, the evaluation was performed on a limited number of developmental stages and two heart datasets, and broader assessment across tissues and biological conditions will be needed. Third, the quality of the inferred trajectory remains dependent on the accuracy of upstream spot-state estimation and on the annotation quality of the single-cell reference. In addition, coarse composition vectors may not capture within-state transcriptional changes that are visible at single-cell resolution.

When high-quality single-cell-resolution spatial transcriptomics data are available, trajectory methods can be applied directly at the cell level and may provide finer-grained information on cell-state transitions. st2traj instead targets the common spot-based setting, such as 10x Visium, in which each observation may contain multiple cell states. In this setting, the deconvolution-derived representation provides an interpretable input for trajectory inference without requiring direct spatial coordinate correspondence across sections.

Future work will extend st2traj to additional tissues, broader benchmark settings, and more flexible trajectory backends, as well as explore its applicability to higher-resolution spatial assays. Overall, st2traj provides a practical and reproducible framework for deconvolution-informed trajectory analysis in multi-timepoint spatial transcriptomics, and highlights the importance of representation design for obtaining spatially coherent and biologically interpretable trajectories.

## Supplementary Material

btag645_Supplementary_Data

## Data Availability

The human heart datasets analysed in this study were obtained from previously published public resources. We used the processed data available from the two Mendeley Data repositories: https://doi.org/10.17632/fhtb99mdzd.1 and https://doi.org/10.17632/w65jtfsvpr.1. Source details for the original dataset are described in the corresponding publication. The developmental chicken heart single-cell RNA-sequencing and spatial transcriptomics data were obtained from the Gene Expression Omnibus under accession GSE149457.

## Code availability

The source code, analysis notebooks, and scripts are publicly available at GitHub (https://github.com/xiaoxiaoxier/st2traj). The exact software snapshot associated with the analyses reported in this manuscript, version 0.1.0, has been archived in Zenodo under DOI https://doi.org/10.5281/zenodo.21487030. Processed data are available in Zenodo under DOI https://doi.org/10.5281/zenodo.21502094.
