# Supplementary material for: st2traj: deconvolution-informed trajectory inference for multi-timepoint spatial transcriptomics
Source: Bioinformatics. 2026 Aug 27;42(9):btag645. doi: 10.1093/bioinformatics/btag645 (PMC13585445; doi:10.1093/bioinformatics/btag645)
Supplement: Supplementary file 1 [file btag645_supplementary_data.pdf]

st2traj: deconvolution-informed trajectory inference for multi-timepoint spatial transcriptomics

Zhuo Wang[1*] and Chiping Zhang[2]

[1]Department of Computational Mathematics and Cybernetics, Shenzhen MSU–BIT University, Shenzhen, Guangdong, 518172, P.R. China
[2]School of Mathematics, Harbin Institute of Technology, Harbin, Heilongjiang, 150000, P.R. China
[*]To whom correspondence should be addressed. E-mail(s): wangzhuo@smbu.edu.cn.

1 Supplementary Results

1.1 Sensitivity of RCTD performance to the assumed mixture mode

Because the simulated pseudo-spots were allowed to contain contributions from multiple cell states, RCTD was run in full mode in the primary pseudo-spot benchmark. Full mode permits unrestricted cell-state mixtures, whereas doublet mode models each spot under a singlet-or-doublet assumption. To assess the influence of this modelling choice, we additionally evaluated RCTD in doublet mode using the identical reference, test pseudo-spots, cell-state definitions, gene space, and evaluation metrics.

RCTD-full achieved an overall concordance correlation coefficient (CCC) of 0.734, a Pearson correlation of 0.746, and a root mean squared error (RMSE) of 0.116. In comparison, RCTD-doublet achieved a CCC of 0.495, a Pearson correlation of 0.583, and an RMSE of 0.212 (Supplementary Fig. S1A). The improvement obtained with full mode was also observed across all seven individual cell-state categories (Supplementary Fig. S1B–D). Particularly large differences were observed for FB/EPDC, SMC/MC/PC, EC, and Neural states.

These results are consistent with the design of the pseudo-spot benchmark. Because a simulated pseudo-spot could contain more than two cell states, the unrestricted full-mixture model was better aligned with the underlying data-generating process than the doublet model. The analysis therefore demonstrates that the strong performance of RCTD in the primary benchmark depends substantially on whether its assumed mixture mode matches the expected complexity of the spatial spots.

Although RCTD-full achieved the highest overall CCC and Pearson correlation in the primary benchmark, its marked dependence on the specified mixture model highlights the importance of appropriate model selection. DECODE, by contrast, does not require the user to choose between doublet and full mixture modes and showed competitive and comparatively balanced performance across the benchmark metrics. Together with its integration into the current end-to-end `st2traj` workflow, this supported the use of DECODE as the default spot-state estimation backend in the present implementation. This choice does not imply that DECODE is universally superior to RCTD or other deconvolution approaches.

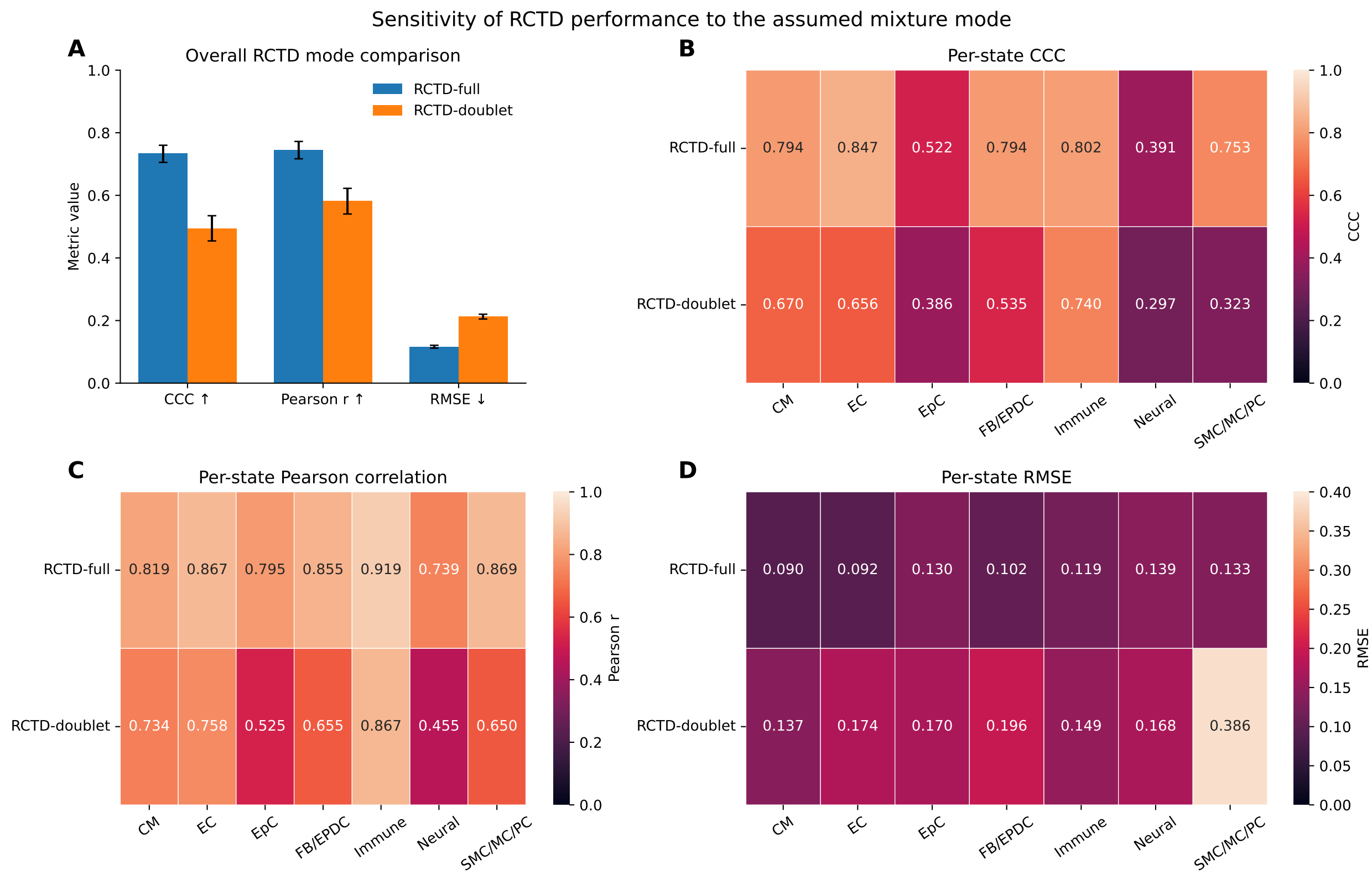

Figure S1: **Sensitivity of RCTD performance to the assumed mixture mode.** RCTD-full and RCTD-doublet were evaluated on the same 300 held-out pseudo-spots. (**A**) Overall concordance correlation coefficient (CCC), Pearson correlation, and root mean squared error (RMSE). Error bars indicate 95% confidence intervals from 2,000 spot-level bootstrap replicates. (**B–D**) Per-state CCC (**B**), Pearson correlation (**C**), and RMSE (**D**) across the seven coarse cell-state categories.

1.2 Spatial organization of DECODE-derived states and internal composition consistency

We next examined the spatial organization of the DECODE-derived spot-state representation in the three developmental human heart sections. Each spot was assigned its dominant state according to the largest estimated DECODE proportion. The resulting spatial maps showed

structured and stage-dependent distributions of the dominant states across the w8, w10, and w12 sections (Supplementary Fig. S2A). These patterns indicate that the DECODE-derived representation preserved spatially organized differences in tissue composition rather than producing spatially unstructured assignments.

We further evaluated the relationship between the oriented trajectory potential and the DECODE-derived state proportions. For each developmental stage, Spearman correlations were calculated between the trajectory potential and each of the seven state proportions. The stage-specific correlations were then averaged across w8, w10, and w12 (Supplementary Fig. S2B,C). Distinct states showed different directions and magnitudes of association with the inferred trajectory potential, indicating that progression along the trajectory was accompanied by coordinated changes in the spot-state representation.

Because both the trajectory representation and the state proportions used in this analysis were derived from DECODE, these correlations should be interpreted as measures of internal representation consistency rather than as independent biological validation. They demonstrate that the trajectory potential varies systematically with the composition features used to construct the manifold, while complementary expression-based assessment is provided separately through expression-derived marker associations and developmental-stage analyses in the main text.

1.3 Expression-derived marker-program assessment of the human heart trajectory

Marker-module scores calculated from normalized spot-level expression using the predefined gene sets in Supplementary Table S1 showed systematic changes along the oriented trajectory. CM-associated expression decreased toward high potential, whereas FB/EPDC- and SMC/MC/PC-associated programs increased. The same patterns were visible in comparisons of low- and high-potential spots and in the corresponding spatial maps.

1.4 Exploratory comparison with moscot

Both st2traj and the moscot-derived OT-informed score captured broad developmental organization, but they represented different properties of the data. The moscot-derived score was spatially smoother and showed stronger associations with four representative raw-expression-derived marker programs, whereas st2traj provided an explicit potential and directional field on the deconvolution-informed state manifold. The comparison was therefore treated as exploratory rather than as a direct ranking of trajectory accuracy.

1.5 Exploratory section-level comparison with CASCAT

In the representative w10 section, both methods produced spatially structured trajectory scores. The `st2traj` potential showed lower local spatial roughness, whereas the overall expression-derived marker-program association was similar between the two methods. Because CASCAT requires selection of a trajectory root and operates at the section level, this analysis was treated as an exploratory comparison.

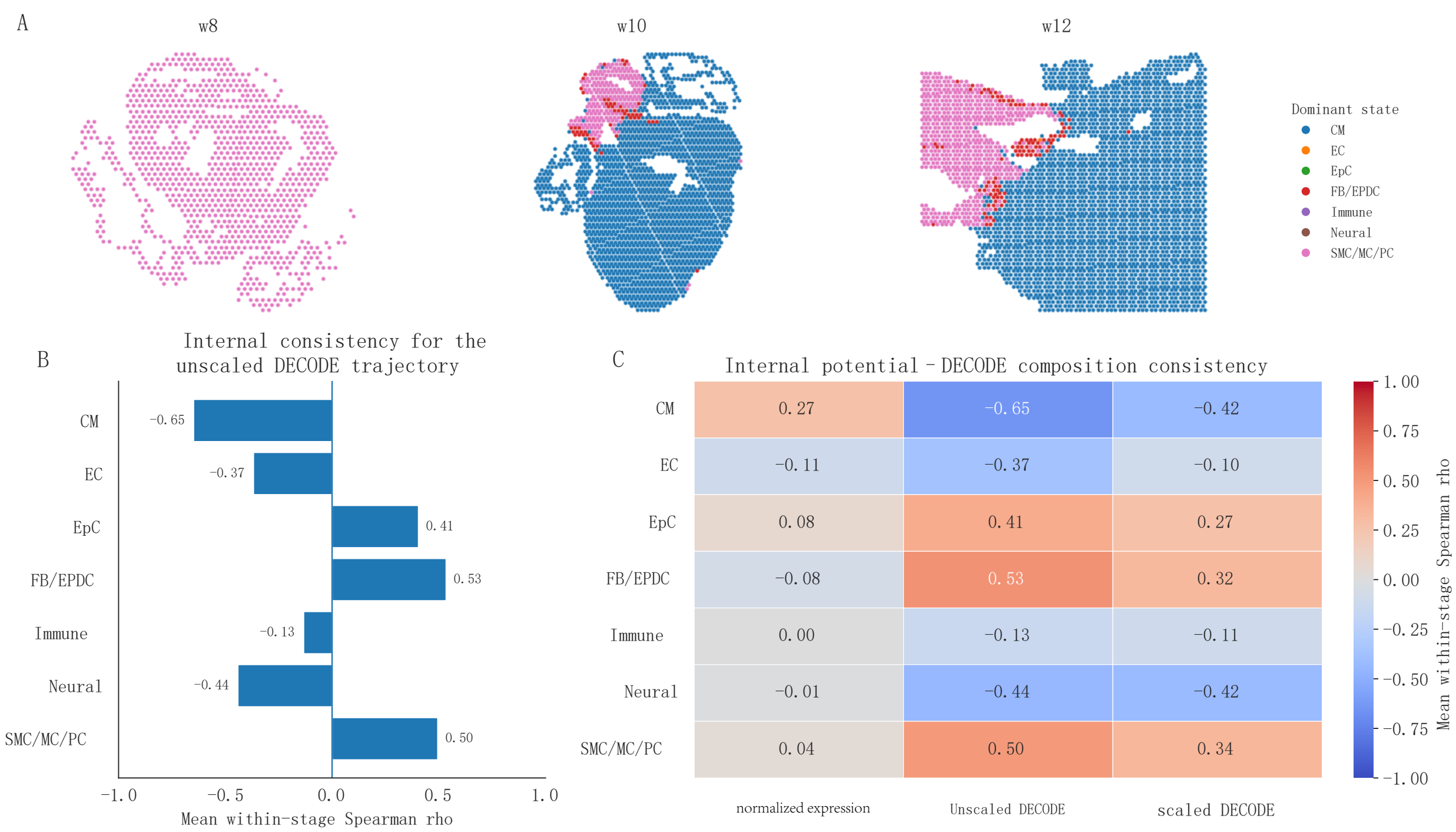

Figure S2: **Spatial organization of dominant DECODE states and internal composition consistency of the inferred trajectory.** (**A**) Spatial distributions of dominant DECODE-derived states in the w8, w10, and w12 human heart sections. (**B**) Mean within-stage Spearman correlations between the oriented potential inferred from unscaled DECODE proportions and the seven DECODE-derived state proportions. (**C**) Corresponding correlations for trajectories inferred from normalized spot-level expression, unscaled DECODE proportions, and scaled DECODE proportions. Panels B and C represent internal composition-consistency assessments rather than independent biological validation.

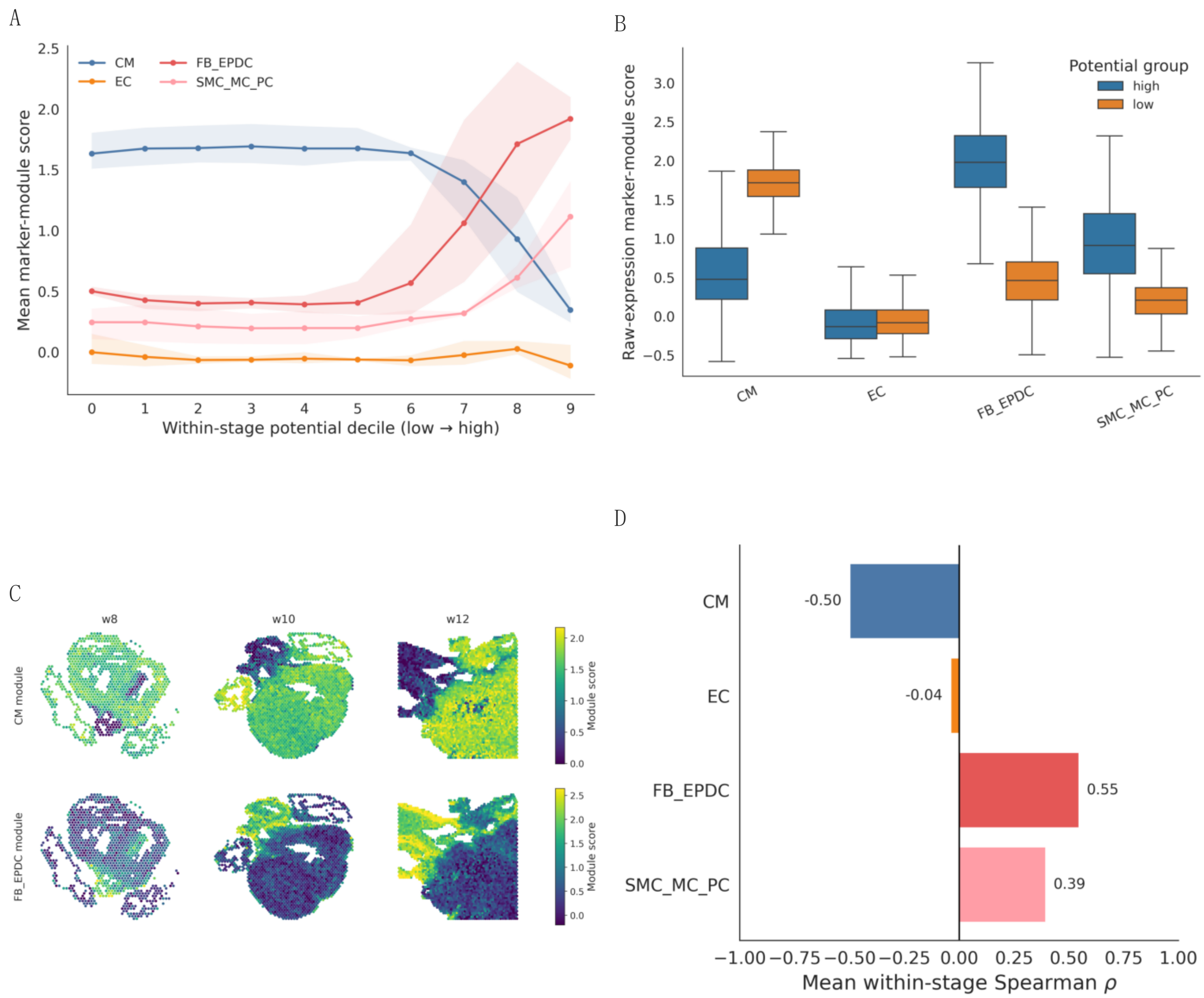

Figure S3: **Expression-derived marker-program assessment of the inferred human heart trajectory.** Marker-module scores were calculated from normalized spot-level expression and were not used as trajectory inputs. (**A**) Mean module scores across within-stage potential deciles; shaded regions show the range across w8, w10, and w12. (**B**) Module-score distributions in the bottom and top 20% of oriented potential within each stage. (**C**) Spatial distributions of representative CM- and FB/EPDC-associated module scores. (**D**) Mean within-stage Spearman correlations between oriented potential and representative marker-module scores.

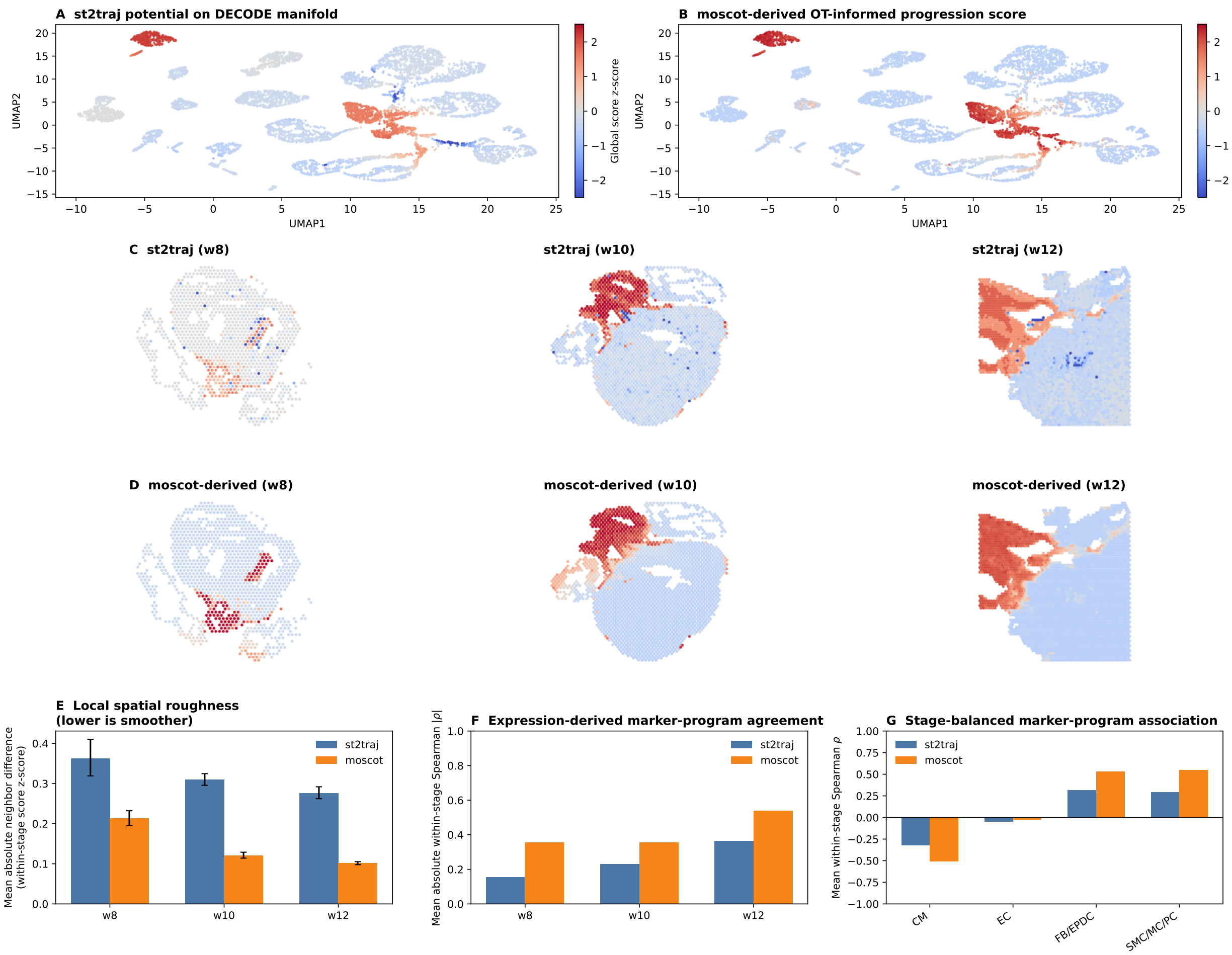

Figure S4: **Exploratory comparison of st2traj and a moscot-derived OT-informed progression score.** (**A**, **B**) Oriented st2traj potential and moscot-derived score on the shared DECODE-derived manifold, shown as global z-scores. The latter is an exploratory summary of transport couplings rather than a native moscot pseudotime. (**C**, **D**) Spatial distributions of the corresponding scores across w8, w10, and w12 after within-stage standardization. (**E**) Local spatial roughness, quantified by the mean absolute neighboring-score difference; error bars indicate 95% edge-bootstrap confidence intervals. (**F**) Mean absolute within-stage correlations with four expression-derived marker programs. (**G**) Stage-balanced signed correlations with the same programs. Marker-program scores were calculated from normalized spot-level expression and were not used as trajectory inputs.

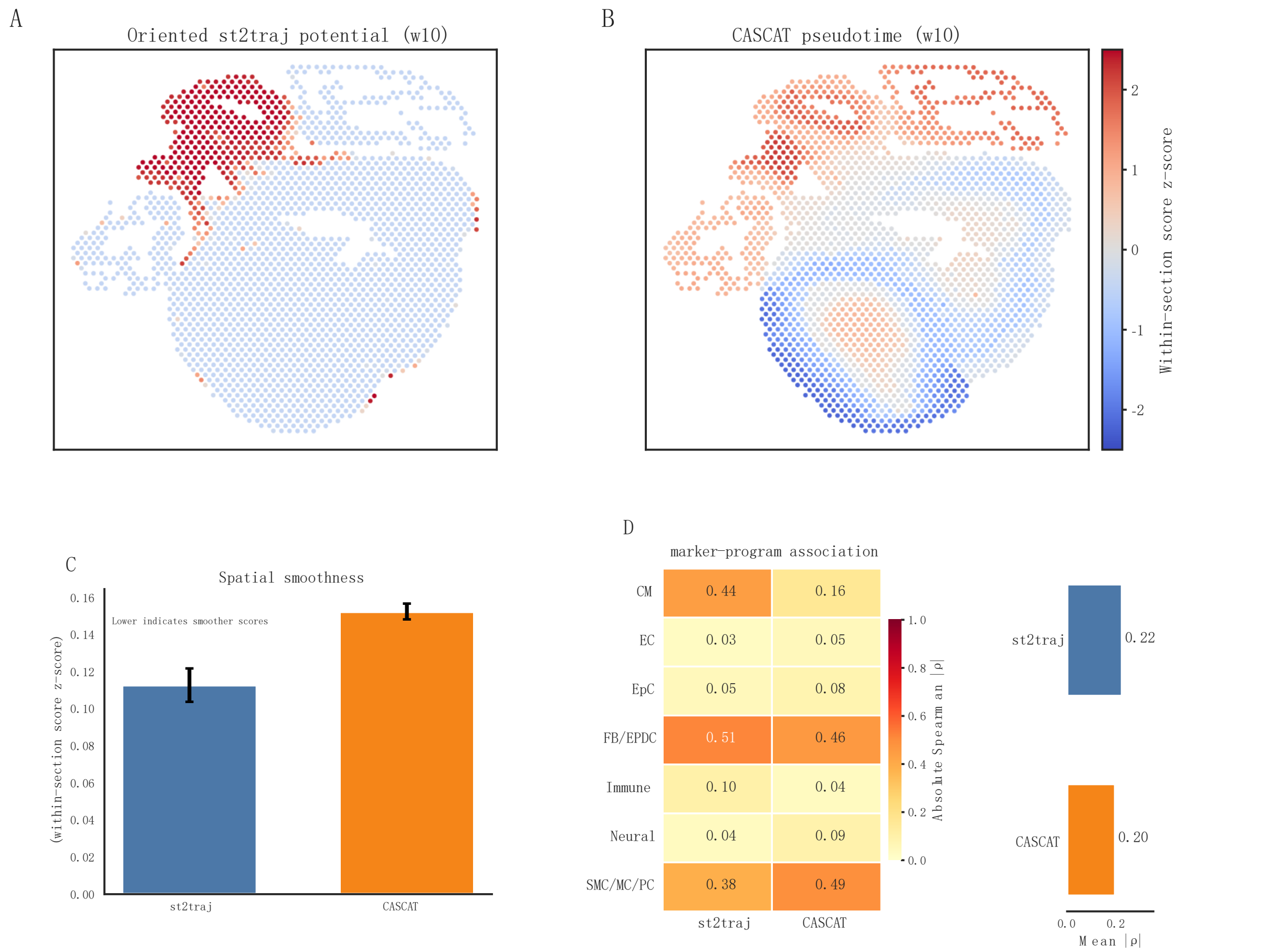

Figure S5: **Exploratory comparison of `st2traj` and CASCAT in the representative w10 human heart section.** (**A**) Spatial distribution of the oriented `st2traj` potential. (**B**) CASCAT pseudotime on the same aligned spots. Scores in panels A and B were standardized within the w10 section and displayed using a common color scale. (**C**) Mean absolute neighboring-score difference on the same six-nearest-neighbor spatial graph. Error bars indicate 95% edge-bootstrap confidence intervals. (**D**) Absolute Spearman correlations with marker-module scores calculated from normalized spot-level expression, together with the mean correlation across the seven programs.

1.6 Cross-species application in developing chicken heart

We further applied `st2traj` to an independent chicken heart spatial transcriptomics dataset spanning D7, D10, and D14. The inferred manifold, directional field, and tissue-level potential maps showed organized trajectory structure. Potential distributions differed across developmental days, although their monotonic association with day was modest. State-specific associations with DECODE-derived proportions provided an internal consistency assessment in this independent cross-species dataset.

Table S1: **Marker genes used to construct expression-derived cell-state modules in the human heart analyses.** Module scores were calculated from normalized spot-level expression. Only genes present in the expression matrix were retained, and a module was evaluated only when at least three marker genes were available. The marker-module scores were not used as trajectory inputs.

**Module**	**Number of genes**	**Marker genes**
CM	9	*TNNT2*, *TNNI3*, *MYH6*, *MYH7*, *ACTC1*, *PLN*, *RYR2*, *ATP2A2*, *CKM*
EC	8	*PECAM1*, *VWF*, *EMCN*, *KDR*, *ENG*, *RAMP2*, *CA4*, *ESAM*
EpC	8	*WT1*, *TCF21*, *TBX18*, *ALDH1A2*, *UPK3B*, *KRT8*, *KRT18*, *KRT19*
FB/EPDC	8	*COL1A1*, *COL1A2*, *COL3A1*, *DCN*, *LUM*, *COL6A1*, *COL6A2*, *FN1*
Immune	8	*PTPRC*, *LST1*, *TYROBP*, *FCER1G*, *C1QA*, *C1QB*, *CD74*, *HLA-DRA*
Neural	8	*S100B*, *SOX10*, *PLP1*, *MPZ*, *SLC1A3*, *FABP7*, *NCAM1*, *GAP43*
SMC/MC/PC	8	*ACTA2*, *TAGLN*, *MYL9*, *RGS5*, *CSPG4*, *MCAM*, *PDGFRB*, *NOTCH3*

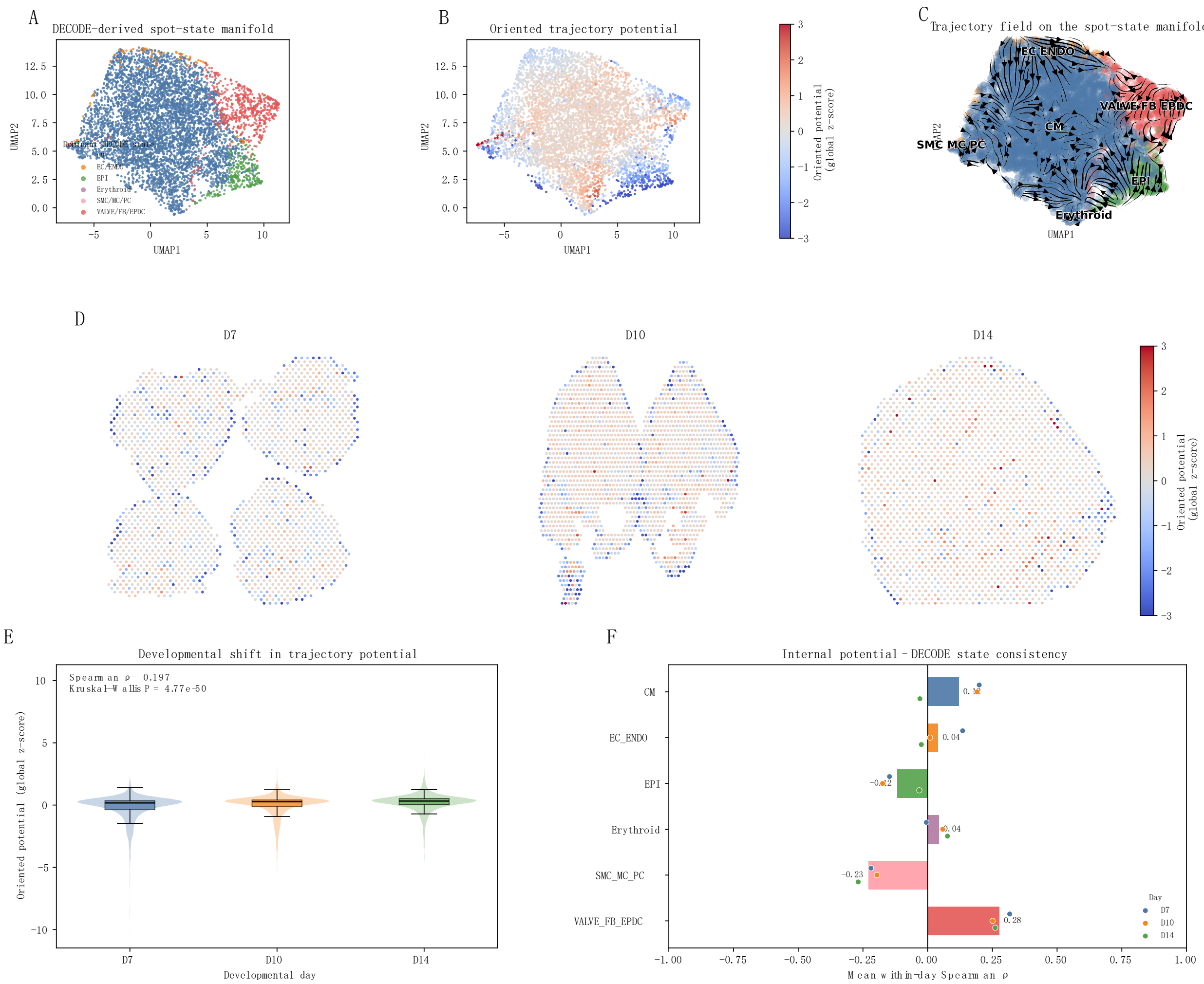

Figure S6: **Cross-species application of `st2traj` to developing chicken heart spatial transcriptomics data.** (**A**) Integrated DECODE-derived spot-state manifold colored by dominant state. (**B**) Oriented trajectory potential on the manifold, shown as a global z-score. (**C**) Potential-derived trajectory field on the spot-state manifold. (**D**) Spatial distributions of globally standardized potential in D7, D10, and D14 sections. (**E**) Potential distributions across developmental days, with the Spearman correlation and Kruskal–Wallis test shown in the panel. (**F**) Mean within-day Spearman correlations between oriented potential and DECODE-derived state proportions; colored points show day-specific correlations. Panel F represents an internal composition-consistency assessment.
